# Mechanisms of dyslipidemia-induced erectile dysfunction: a narrative review

**DOI:** 10.3389/fendo.2026.1780243

**Published:** 2026-04-22

**Authors:** Dicheng Luo, Di Sun, Elena Colonnello, Li-Ping Pan, Shuang Wu, Jun Guo, Hao Wang, Jiwei Zhang

**Affiliations:** 1 Graduate School, Beijing University of Chinese Medicine, Beijing, China; 2 Department of Andrology, Xiyuan Hospital, China Academy of Chinese Medical Sciences, Beijing, China; 3 Endocrinology and Medical Sexology(ENDOSEX), Department of Systems Medicine, University of Rome Tor Vergata, Rome, Italy

**Keywords:** cholesterol, dyslipidemia, endocrine, erectile dysfunction, mechanism, vascular

## Abstract

**Background:**

Dyslipidemia is strongly associated with obesity, metabolic syndrome, and atherosclerosis, and is increasingly recognized as an independent risk factor for erectile dysfunction (ED). Emerging evidence suggests lipid-specific mechanisms that may directly impair erectile function, beyond the effects of obesity.

**Objective:**

To summarize the pathophysiological mechanisms linking dyslipidemia to the development and progression of ED.

**Methods:**

A narrative review of studies published between 2000 and 2025 was conducted using PubMed, Web of Science, Scopus, and Google Scholar, including experimental, clinical, observational, and review studies.

**Results and conclusion:**

Dyslipidemia contributes to ED through multiple interconnected pathways, including vascular dysfunction, neural impairment, oxidative stress, inflammation, and endocrine alterations. These mechanisms may increase susceptibility to ED in affected patients. However, direct evidence remains limited, and confounding by coexisting metabolic, cardiovascular and neural conditions complicates interpretation. Further research is needed to clarify the independent effects of dyslipidemia and to identify potential therapeutic strategies.

## 1 Introduction

Dyslipidemia refers to a pathological state where the concentration of one or more lipid components in serum falls outside the upper and lower limits of the normal reference range, and it is recognized as one of leading but modifiable risk factors for the development of atherosclerotic cardiovascular disease (ASCVD) and endothelial dysfunction (1). Its core features are abnormally elevated levels of serum total cholesterol (TC), low-density lipoprotein cholesterol (LDL-C), and triglycerides (TG), as well as pathologically reduced high-density lipoprotein cholesterol (HDL-C), a combination frequently seen in clinical settings (2). Clinically, this metabolic disorder is mainly classified into four standardized phenotypes: isolated hypercholesterolemia (elevated serum TC and/or LDL-C), isolated hypertriglyceridemia (markedly elevated serum TG), mixed dyslipidemia (concurrently elevated serum TC/LDL-C and TG), and isolated hypoalphalipoproteinemia (isolated reduction in serum HDL-C) (3). Due to its high prevalence, it represents a substantial burden to health systems and the economy worldwide. According to a World Health Organization (WHO) estimate, the global prevalence of raised plasma total cholesterol levels among adults aged ≥25 years was approximately 39% in 2008 (2). Furthermore, a 2025 global systematic review and meta-analysis estimated the global prevalence of hypercholesterolemia, hypertriglyceridemia, low HDL-C, and high LDL-C at 24.1%, 28.8%, 38.4%, and 18.93%, respectively, with marked regional variation (4). Driven by adverse global shifts in lifestyle and dietary structure, both the prevalence and incidence of dyslipidemia are increasing annually, particularly among younger populations (5).

Erectile dysfunction (ED) is defined as the persistent or recurrent inability to achieve and maintain a sufficient penile erection to complete satisfactory sexual intercourse (6). It is a growing male health problem worldwide (7). Epidemiological studies show that the overall prevalence of ED in men over 40 is approximately 20%-40% (8), increasing significantly with age, reaching up to 50% by age 70 (9). Notably, ED is no longer considered merely a condition of the elderly, as its incidence in younger populations is also rising, closely related to metabolic disorders caused by modern lifestyles (10). Among these metabolic conditions, dyslipidemia has emerged as a particularly important factor.

**Figure 1:**
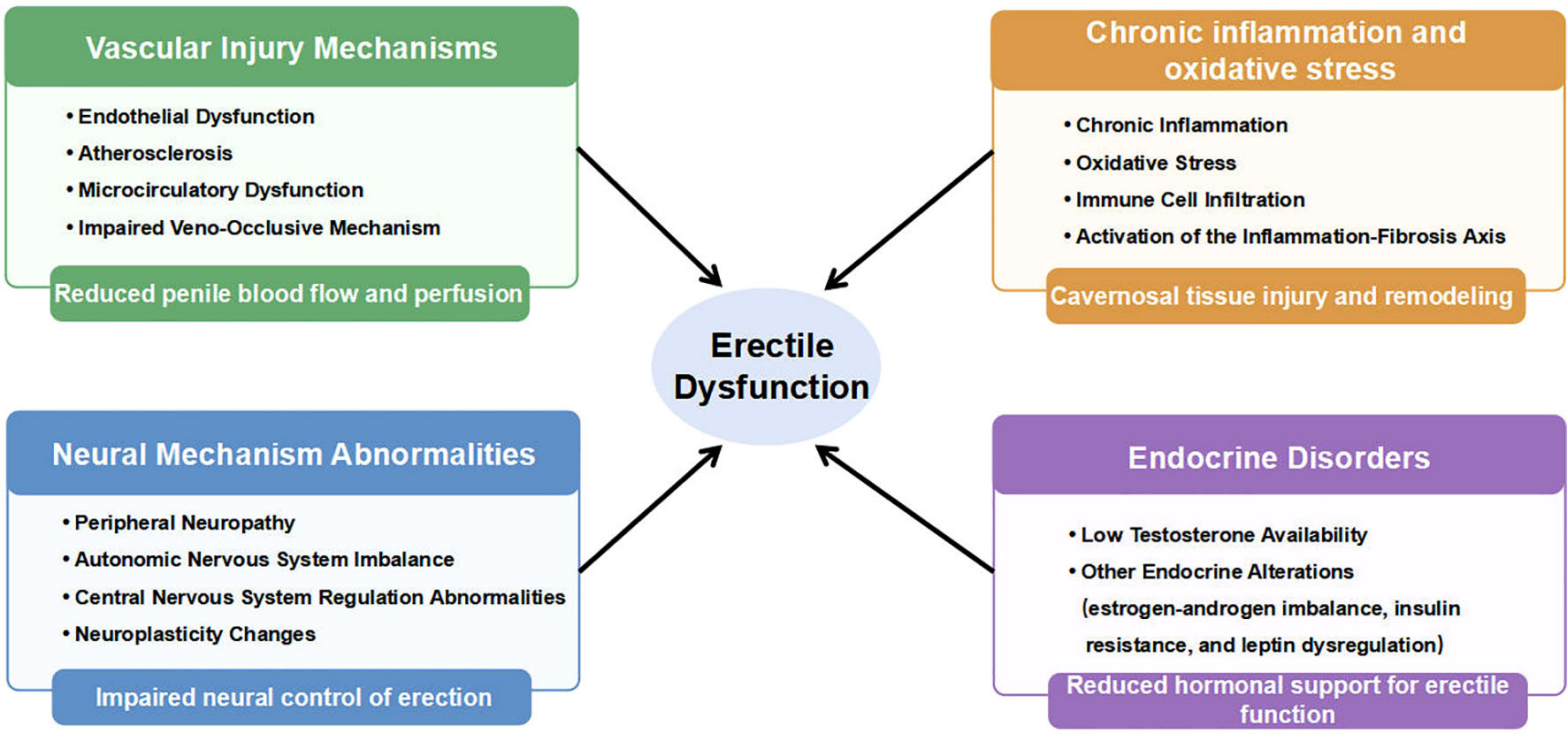
Potential mechanisms linking dyslipidemia to ED. A conceptual overview of the major mechanistic domains discussed in this review.

Many studies indicate a significant correlation between dyslipidemia and ED (11). Although dyslipidemia frequently coexists with obesity and metabolic syndrome, research suggests the existence of lipid-specific mechanisms that may directly impair penile vascular and neural function (11). Population-based analyses have also reported associations between dyslipidemia-related metabolic indices and ED (12, 13). Therefore, this review aims to outline the multi-mechanism theoretical framework of dyslipidemia-associated ED (**Figure 1**).

## 2 Methods

The research strategy followed in producing this article is that of a narrative review, which was used to gather and synthesize evidence on dyslipidemia-induced ED. We searched PubMed, Web of Science, Scopus, and Google Scholar for English-language publications from 2000 until December 2025, using keywords related to dyslipidemia (“dyslipidemia”, “hyperlipidemia”, “hypercholesterolemia”, “hypertriglyceridemia”, “low HDL”, “hypoalphalipoproteinemia”) combined with terms for ED (“erectile dysfunction”, “impotence”, “erection mechanisms”).

In contrast to a systematic review, the study selection for this narrative review was not exhaustive but prioritized based on relevance, novelty, and mechanistic insight. Articles of experimental studies, clinical observational studies, meta-analyses, and high-quality narrative or systematic reviews were first selected. Case reports and studies without mechanistic relevance were generally excluded.

Focusing on studies that explore the dyslipidemia-ED association, we critically appraised and synthesized the selected literature to construct a cohesive conceptual framework. Rather than conducting a formal meta-analysis, we thematically organized the evidence into four key pathogenic domains: Vascular Injury, Endocrine Disturbances, Neurological Mechanisms, and the Interplay of Chronic Inflammation and Oxidative Stress. This integrative process followed academic standards to ensure a logical and structured evidence presentation.

Notably, this narrative review provides a comprehensive overview of possible biologically plausible mechanisms potentially linking dyslipidemia to ED, rather than focusing exclusively on studies with direct causal evidence obtained from ED-specific experimental or clinical research. The included evidence integrates findings from both systemic and ED-specific studies to develop a theoretical framework of potential pathogenic mechanisms.

## 3 Physiological basis of normal erection

**Figure 2:**
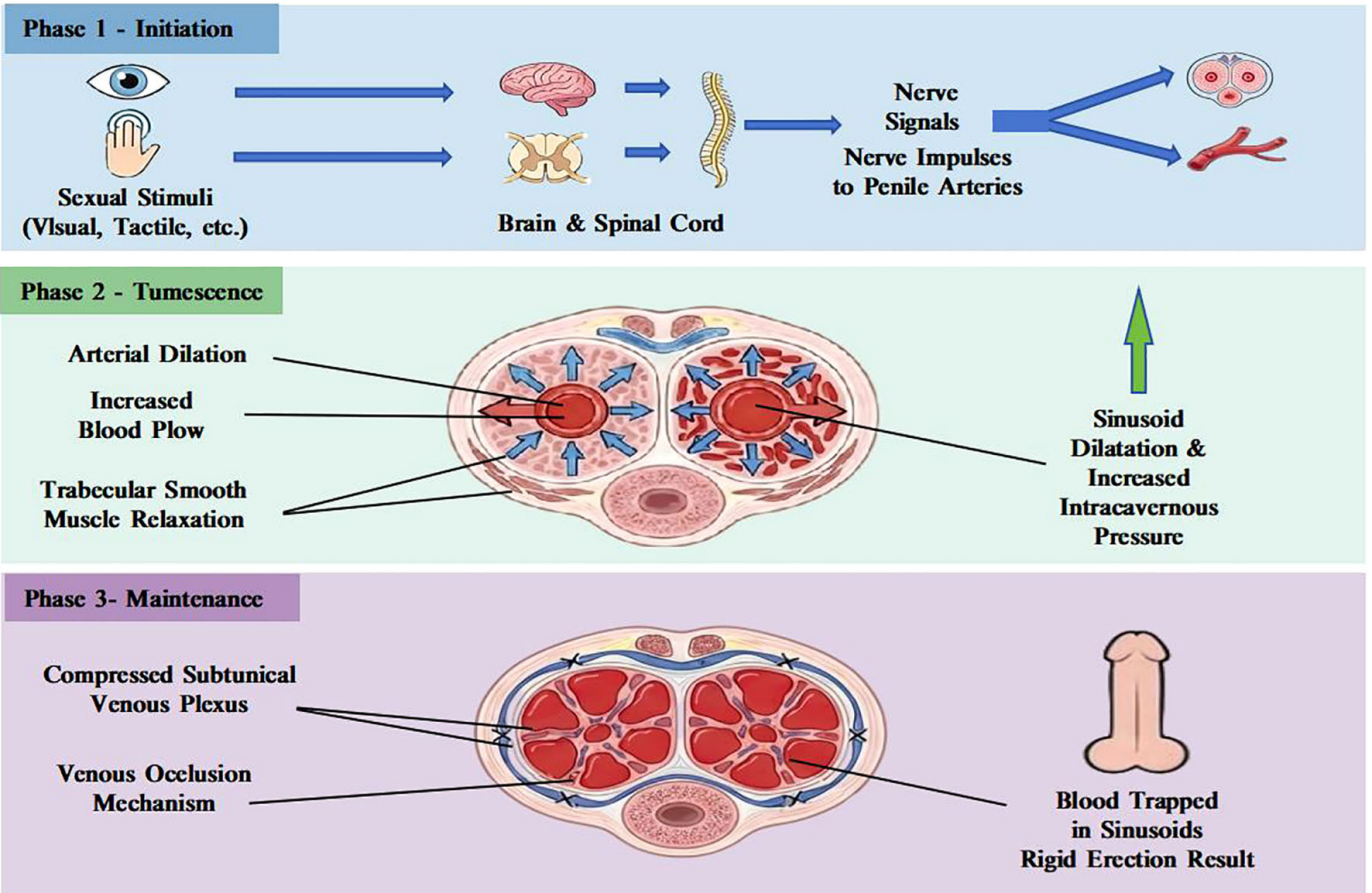
Normal erectile physiological mechanism.

Penile erection is a complex neurovascular event dependent on precisely regulated hemodynamic changes and molecular signaling (14). The erection process can be divided into three phases: initiation, tumescence, and maintenance (15),each involving unique physiological mechanisms (**Figure 2**). Erection initiation begins with sexual stimuli (visual, tactile, or imaginative) transmitted via afferent nerves to the spinal cord and related brain regions, followed by efferent signals from the autonomic nervous system (primarily parasympathetic). These nerve signals promote dilation of the penile cavernous arteries, a sharp increase in blood flow, and relaxation of the trabecular smooth muscle and dilatation of the sinusoids. As the intracavernous pressure rises, the subtunical venous plexus is compressed, forming a venous occlusion mechanism that traps blood within the penis, ultimately resulting in a rigid erection (16).

At the molecular level, the nitric oxide (NO)-cyclic guanosine monophosphate (cGMP) signaling pathway is the core mechanism mediating erection (17, 18). Nitric oxide (NO) plays a key role in regulating vasodilation, arterial blood pressure and penile erection. Endothelial nitric oxide synthase (eNOS) is the key enzyme for NO production, and its activity is regulated by various factors, including blood flow shear stress, hormone levels, and oxidative stress status (19, 20). Furthermore, substances such as L-arginine and L-citrulline can increase NO production, supporting sexual function and vascular health (21). Nerve impulses promote the release of NO from endothelial cells. NO activates guanylate cyclase, increasing intracellular cGMP levels, leading to relaxation of cavernous smooth muscle, thereby increasing penile blood flow and achieving erection.

## 4 Vascular injury mechanisms

### 4.1 Endothelial dysfunction

Endothelial dysfunction is the possible primary trigger in dyslipidemia-induced impairment of erectile function. Indeed, dyslipidemia can lead to impaired penile endothelial function, thereby affecting erection, through multiple mechanisms: oxidation of low-density lipoprotein (LDL) leading to stimulation of endothelial cells to release endothelin and reactive oxygen species (ROS), which further reduce NO bioavailability (22); increase of oxidative stress in endothelial cells, leading to inactivation of eNOS, thereby reducing NO production (23). Additionally, postprandial dyslipidemia also impairs endothelial function. High-fat diet-induced obese rats showed reduced endothelium-dependent relaxation of penile arteries in response to acetylcholine and the mitochondrial ATP-sensitive potassium channel activator BMS191095, indicating impaired endothelial function and a decreased ability of endothelial cells to synthesize and release nitric oxide (NO), thus reducing NO bioavailability (24, 25).

In a study of 40 premenopausal women and 40 age- and BMI-matched men (26), the number and activity of circulating endothelial progenitor cells (EPCs-bone marrow-derived cells involved in vascular repair and endothelial homeostasis) and NO levels in plasma or culture medium were significantly reduced in dyslipidemic men. In contrast, EPC number and activity, as well as NO production, were preserved in premenopausal hypertriglyceridemic women (26). These findings suggest a role for sex differences in hypertriglyceridemia-associated vascular injury, which may impair erectile function via the nitric oxide-cyclic guanosine monophosphate (NO-cGMP) signaling pathway. Furthermore, these changes can reduce vasodilatory capacity, thereby affecting normal blood flow regulation and erectile function (27).In a prospective randomized trial, 10 subjects treated for 4 weeks with ezetimibe -a selective cholesterol absorption inhibitor that blocks intestinal cholesterol uptake by targeting the Niemann-Pick C1-like 1 (NPC1L1) transporter-showed significant attenuation of postprandial elevations in triglycerides, remnant lipoprotein cholesterol, and apolipoprotein B-48 levels, along with improvement in postprandial endothelial function, as assessed by brachial artery flow-mediated dilation (28).

### 4.2 Atherosclerosis

Dyslipidemia, particularly LDL-C elevation, causes lipid deposition in the arterial wall, triggering plaque formation (29). Studies showed that the LDL/HDL ratio is negatively correlated with the peak systolic velocity (PSV) of the cavernous artery, while HDL-C is positively correlated with PSV (30). In patients with dyslipidemia, this leads to hemodynamic changes in the penile arteries, shown by ultrasound as a significant decrease in cavernous artery PSV and an increase in resistance index (RI), reflecting arterial inflow dysfunction, thereby affecting erectile function (31). Pathological studies further confirmed that in dyslipidemia animal models, the number of small arteries in penile tissue decreases, the vascular wall thickens, and the lumen narrows (32). In men with coronary artery disease (CAD), the incidence of ED is higher, and the severity of CAD is associated with an increased risk of ED. In a study of 316 CAD patients, about 55.1% had ED. Multivariate analysis showed that CAD severity was an independent risk factor for ED (OR = 4.11, 95% CI = 1.69 - 9.97). The greater the number of affected coronary arteries and the degree of stenosis, the higher the risk of ED (OR = 3.74, 95% CI = 1.72 - 8.09). Age (OR = 1.23, 95% CI = 1.18 - 1.29) and BMI (OR = 1.26, 95% CI = 1.13 - 1.41) were also independent predictors of ED (33).

The penile cavernous arteries are small in diameter (about 0.3-0.5 mm) and particularly susceptible to atherosclerosis. Therefore, based on the association between atherosclerosis and ED, researchers have also proposed that ED can be an early marker of systemic atherosclerosis (34).

### 4.3 Microcirculatory dysfunction

The penile corpus cavernosum is rich in a microvascular network, the integrity of which is crucial for maintaining erectile function. Hypercholesterolemia, an important component of dyslipidemia, can lead to decreased capillary density, reduced expression of vascular endothelial growth factor (VEGF), and abnormal vascular remodeling (35). Concurrently, downregulation of angiogenic factors and their downstream signaling molecules can further exacerbate ED (36). Compared to normal rats, diabetic dyslipidemic rats showed a 37% reduction in microvascular branches and a 19% reduction in microvascular length density (37). Studies have also found that Angiopoietin-1 (Ang1), an angiogenic growth factor, can promote the generation of stable and functional vasculature and rescue erectile function in hypercholesterolemic mice (38).

### 4.4 Impaired veno-occlusive mechanism

During erection, venous occlusion allows blood to accumulate within the corpus cavernosum, a process essential for maintaining penile rigidity. Dyslipidemia may impair veno-occlusive function through both direct structural remodeling of the corpus cavernosum and indirect hemodynamic effects (39–41). On the one hand, dyslipidemia promotes atherosclerosis, reducing penile arterial inflow; the resulting arterial insufficiency may prevent an adequate rise in intracavernosal pressure (41, 42). In addition, chronic arterial insufficiency can induce cavernosal hypoxia and ischemia, which promote progressive tissue remodeling and ultimately impair veno-occlusive function (41).Experimental evidence from hypercholesterolemic rabbit models has demonstrated endothelial and smooth muscle dysfunction in the corpus cavernosum, along with ultrastructural abnormalities in smooth muscle cells, elastic fibers, and collagen, indicating disruption of the structural basis required for effective venous occlusion (39). Long-term hypercholesterolemia has also been shown to cause chronic cavernosal tissue injury that may be only partially reversible after lipid normalization (40).

On the other hand, direct clinical evidence linking dyslipidemia to venogenic ED remains limited. However, interventional studies support the central role of the veno-occlusive mechanism in maintaining erectile function. In patients with mixed arteriovenous ED who did not benefit from arterial revascularization alone, additional venous leak embolization significantly improved outcomes. Only 11.5% (3/26) of patients achieved the primary feasibility endpoint (an increase in IIEF-5 score ≥ 4 points) 6 weeks after arterial revascularization alone, whereas this proportion increased to 65.4% (17/26) following additional venous leak embolization (43). Furthermore, in a cohort of 50 patients with severe ED secondary to venous leakage treated with venous embolization, 68% (34/50) reached the primary feasibility endpoint at 6-week follow-up, further confirming the importance of the veno-occlusive mechanism in erectile function (44).Taken together, these findings underscore the importance of intact veno-occlusive function in maintaining penile rigidity, although they do not establish dyslipidemia as a direct cause of venous leakage. Nevertheless, impaired veno-occlusive function may contribute to reduced erection hardness and shorter erection duration in patients with dyslipidemia-associated ED.

## 5 Chronic inflammation and oxidative stress

Dyslipidemia contributes to the pathogenesis of ED through a deeply integrated pathway of chronic inflammation and oxidative stress (45, 46). The process begins with the oxidation of elevated LDL to form oxidized LDL (ox-LDL) (47). Acting as a damage-associated molecular pattern, ox-LDL not only triggers an innate immune response but also stimulates excessive production of reactive oxygen species (ROS), creating a pathogenic feed-forward loop (48). This interplay critically undermines vascular endothelial function and reduces NO bioavailability. As the pathology advances, immune cell infiltration and the activation of the inflammation-fibrosis axis drive irreversible structural fibrotic remodeling of the corpus cavernosum (46).

### 5.1 Chronic inflammation

Low-grade chronic inflammation is one of the possible underlying mechanisms by which dyslipidemia induces ED (49). Studies indicated that inflammation is closely related to ED. In a study of US adults, the Dietary Inflammatory Index (DII) was positively correlated with ED. After adjusting for age, race, education level, and other factors, the odds ratio for the association between DII score and ED was 1.12, indicating a link between the inflammatory potential of diet and ED (50). Another study found that systemic inflammation indicators such as the multi-inflammatory index 1 (MII-1) and MII-2 were significantly higher in the ED group than in the non-ED group and were negatively correlated with the International Index of Erectile Function (IIEF) (51).

In an asthma model in rats, inflammatory factors damaged vascular endothelial cells, leading to ED. Treatment with the traditional Chinese medicine Xuefu Zhuyu Decoction (XFZYD) could inhibit inflammation, increase the number of penile erections, and improve erectile function, while reducing the expression levels of inflammatory factors such as interleukin-6 (IL-6), vascular endothelial growth factor A (VEGFA), and tumor necrosis factor (TNF) in penile tissue. This indicates that chronic inflammation primarily affects erectile function by damaging vascular endothelial cells, and inhibiting inflammation can improve this condition (52).

### 5.2 Oxidative stress

Oxidative stress is an important possible mechanism in dyslipidemia-induced ED. Under normal physiological conditions, the body’s oxidation and antioxidant systems remain balanced. However, dyslipidemia can lead to increased oxidative stress, producing excessive ROS that damage penile tissue. Studies showed that in dyslipidemic rat models, ROS levels in penile cavernous tissue are elevated (53).

Oxidative stress can affect erectile function through multiple pathways. On one hand, ROS can damage vascular endothelial cells, causing endothelial dysfunction and reducing the production and release of NO. In studies on diabetic ED rats, oxidative stress led to decreased expression of eNOS in penile cavernous tissue and reduced NO production. Administration of the antioxidant hesperidin activated the Nrf2-HO-1/GPX4 axis, inhibited oxidative stress and ferroptosis, and improved erectile function (54). Furthermore, oxidative stress can induce apoptosis of penile cavernous smooth muscle cells or sympathetic overactivity and downregulation of soluble guanylate cyclase (55, 56), disrupting the tissue structure and function related to erection.

### 5.3 Immune cell infiltration

Immune cell infiltration is involved in dyslipidemia-related ED. Dyslipidemia is associated with monocyte/macrophage infiltration and activation in penile tissue, which may enhance local inflammatory injury through the release of pro-inflammatory mediators and downstream effectors, including matrix metalloproteinases (MMPs) (57). MMPs may further promote cavernosal remodeling by disturbing extracellular matrix turnover (58). Macrophages may acquire a pro-inflammatory M1 phenotype, which amplifies local inflammatory responses (59) and promotes endothelial dysfunction and impaired penile perfusion.

Experimental studies have shown that macrophage-specific TLR9 signaling is associated with cavernosal dysfunction in high-fat diet-fed C57BL/6 mice (60). In addition, high-fat diet-induced ED models have shown activation of the IL-18/NLRP3/caspase-1 pathway together with reduced eNOS expression (61). Hypercholesterolemic ApoE−/− mice fed a Western diet also showed corpus cavernosum remodeling accompanied by altered MMP-related structural changes (58). Overall, these findings suggest that dyslipidemia-associated monocyte/macrophage infiltration and inflammatory activation may participate in the progression of ED.

### 5.4 Activation of the inflammation-fibrosis axis

Activation of the inflammation-fibrosis axis leads to irreversible structural changes in the corpus cavernosum (62). Mendelian randomization studies have suggested that dyslipidemia is associated with an elevated risk of ED, in which inflammatory mediators serve as a critical contributor (63). In the corpus cavernosum, dyslipidemia activates two synergistic inflammatory pathways. First, oxidized low-density lipoprotein (ox-LDL) induces oxidative stress and activates the nuclear factor-κB (NF-κB) signaling pathway (48, 64, 65), leading to endothelial dysfunction and promoting macrophage-mediated inflammatory changes in cavernosal tissue (60); Second, lipid overload activates the NLRP3 inflammasome and maintains a state of meta-inflammation (61).

Persistent inflammatory stimuli activate fibroblasts and myofibroblasts, resulting in excessive extracellular matrix deposition (58). The inflammatory cytokine IL-17A reprograms corpus cavernosum smooth muscle cells (CCSMCs) via the mTORC2–ACACA pathway, enhancing lipid synthesis and the secretion of fibromatrix proteins (66); transforming growth factor-β (TGF-β) drives the differentiation of fibroblasts into myofibroblasts and promotes collagen production (66); tumor necrosis factor-α (TNF-α) amplifies TGF-β signaling and facilitates CCSMC apoptosis (67). The consequent collagen accumulation impairs CCSMC compliance and disrupts veno-occlusive function, thereby leading to refractory ED (68, 69). Collectively, these findings indicate that the inflammation–fibrosis axis represents a central driver in the pathogenesis of dyslipidemia-induced ED and a promising therapeutic target (70, 71).

## 6 Neural mechanism abnormalities

### 6.1 Peripheral neuropathy

Dyslipidemia may cause various forms of damage to the peripheral nervous system, including direct axonal injury, myelin loss, and progressive conduction deficits (72, 73). Elevated LDL-C, particularly its oxidized form (oxLDL), may promote oxidative stress and inflammation in neurons and Schwann cells (74). In addition, oxidized lipids may contribute to myelin abnormalities and impaired nerve function (72, 73). Elevated triglyceride (TG) levels may alter membrane lipid composition and lipid raft organization, thereby impairing neuronal signal transduction and slowing nerve conduction velocity (73, 75).

The specific mechanisms may involve disorders of neural lipid metabolism and mitochondrial function, which may damage peripheral nerves, slow nerve conduction velocity, and induce nerve fiber damage, thereby potentially contributing to the development of ED (73, 74, 76). However, direct ED-specific evidence linking dyslipidemia to peripheral neuropathy remains limited.

### 6.2 Autonomic nervous system imbalance

Dyslipidemia can contribute to autonomic dysregulation, which may negatively affect erectile function. In healthy young men, acute experimental hyperlipidemia has been shown to reduce baroreflex sensitivity and impair cardiovascular reflexes (77). Clinical evidence also indicates that disordered lipid metabolism is linked to cardiac autonomic dysfunction (78, 79). In patients with recent-onset type 2 diabetes, higher plasma lipid metabolites have been linked to early cardiac autonomic dysfunction (78).

Moreover, hypertriglyceridaemia has been associated with small nerve fibre damage and cardiac autonomic dysfunction even in individuals without diabetes (79). Experimental diet-induced metabolic models similarly demonstrate impaired baroreflex sensitivity (80). Because penile erection depends on parasympathetic-mediated vasodilation and is counteracted by sympathetic activation, dyslipidemia-related autonomic dysregulation may contribute to ED by promoting vasoconstriction and reducing penile perfusion (81).

### 6.3 Central nervous system regulation abnormalities

The hypothalamic preoptic area, limbic system, and cerebral cortex are the primary centers for erection. These regions regulate the spinal erection centers by integrating sensory and emotional inputs. The hypothalamus is especially important for controlling systemic metabolism and reproductive function.

Studies in animal models show that a high-calorie environment activates hypothalamic CD4+ T cells and microglia, promotes macrophage infiltration, and reduces hypothalamic regulatory T cells (Tregs), thereby exacerbating hypothalamic immune activation (82). Dyslipidemia-related metabolic stress, particularly during high-fat feeding, rapidly7 induces hypothalamic inflammation. In rodents, inflammatory signaling emerges within 1–3 days of high-fat diet exposure, followed by reactive gliosis and markers of neuronal injury within the first week; similar mediobasal hypothalamic gliosis has also been documented in humans with obesity (83). Because the medial preoptic area and paraventricular nucleus, together with dopaminergic circuits, are central to sexual behavior and penile erection (84, 85), hypothalamic injury may impair the central integration of sexual stimuli and weaken descending input to the spinal erection centers. Consistently, dopamine release in the medial preoptic area rises before and during copulation, and electrical stimulation of this region elicits intracavernous pressure responses in rats, whereas the paraventricular nucleus is a critical node in erectile control (84–86). Thus, central nervous system dysregulation may represent an important mechanism by which dyslipidemia contributes to erectile dysfunction.

### 6.4 Neuroplasticity changes

Neuroplasticity describes the nervous system’s capacity to remodel its structure and function in response to stimuli, including neurogenesis, axonal regeneration, and synaptic remodeling, which is critical for sustaining the neural pathways mediating penile erection. Studies have demonstrated that dyslipidemia may directly disrupt intracavernosal neuroplasticity: it downregulates the intracavernosal expression of nerve growth factor (NGF) and vascular endothelial growth factor (VEGF), two core mediators of neural repair, an effect that impedes neural repair processes and reduces the regenerative capacity of nerve fibers (87–89), thereby impairing erectile-related neural signal transduction and contributing to the onset and progression of ED.

Furthermore, evidence indicates that dyslipidemia is significantly associated with neural aging-related phenotypes and reduced neuroplasticity, which can further impair the function of the cavernous nerve plexus and central erectile regulatory circuits, serving as a potential indirect pathway for dyslipidemia to impair erectile function (90, 91).However, neuroplasticity’s specific role in dyslipidemia-associated ED remains to be elucidated and warrants further investigation.

## 7 Endocrine disorders

### 7.1 Low testosterone availability

Numerous clinical studies indicate that dyslipidemia can affect testosterone levels through various pathways: a high-cholesterol diet can induce endoplasmic reticulum stress in the testes, thereby inhibiting the expression of steroidogenic enzymes and leading to decreased testosterone levels (92). Inflammatory factors may also affect testosterone synthesis by inhibiting the function of the hypothalamic-pituitary-gonadal (HPG) axis (93).

Studies have found that oxidized low-density lipoprotein (oxLDL) can inhibit testosterone synthesis in Leydig cells by affecting mitochondrial function and the p38 MAPK/COX-2 signaling pathway, thereby reducing testosterone availability and leading to ED (94). In a multicenter cross-sectional study of 411 patients (mean age 63.19 years), 91.73% had varying degrees of ED. Total testosterone levels decreased from a median of 7.05 ng/mL in patients with normal erectile function to 3.56 ng/mL in patients with severe symptoms, and free testosterone levels gradually decreased, with the median free testosterone level in all ED groups below the normal lower threshold (95). In addition, studies on ED patients found that parameters in the testosterone-deficient group, such as the number of nocturnal erections per night, average event rigidity, erection duration, and percentage of erection exceeding baseline in the nocturnal penile tumescence and rigidity (NPTR) test, were significantly lower than those in the normal testosterone group (96).

Dyslipidemia may also contribute to testosterone deficiency through modulation of the gut microbiota, particularly via enrichment of testosterone-degrading species (97). In male patients with dyslipidemia, the relative abundance of Pseudomonas nitroreducens, a Gram-negative intestinal commensal capable of metabolizing testosterone, is significantly higher than in controls (98). This bacterium expresses the 3/17β-hydroxysteroid dehydrogenase (3/17β-HSD) gene, which encodes a key enzyme involved in testosterone catabolism. Specifically, 3/17β-HSD catalyzes the oxidation of hydroxyl groups in the testosterone molecule, converting biologically active testosterone into inactive androgen metabolites, thereby reducing circulating testosterone levels (98, 99). The presence of this gene has been associated with both testosterone degradation and dyslipidemia, suggesting that intestinal testosterone-degrading bacteria may contribute to the pathogenesis of dyslipidemia-associated ED through reduced testosterone availability (97, 99). However, whether this microbial alteration is attributable to dyslipidemia itself or to coexisting obesity-related metabolic disturbances remains uncertain, and the independent contribution of dyslipidemia requires further clarification.

### 7.2 Other endocrine alterations

Other endocrine and metabolic alterations, including estrogen-androgen imbalance, insulin resistance, and leptin dysregulation, may coexist in patients with dyslipidemia. However, current evidence does not establish a direct causal link between these changes and dyslipidemia itself, as they are more commonly associated with obesity and related metabolic disturbances. Therefore, these mechanisms should be interpreted cautiously, and their contribution to dyslipidemia-associated ED remains uncertain.

## 8 Interaction among mechanisms

Dyslipidemia-induced ED arises not from isolated pathogenic mechanisms but from an integrated network of vascular, endocrine, neural, and immunometabolic disturbances that mutually reinforce one another (100). Central to this network is endothelial injury, which arises from the accumulation of circulating lipids, particularly low-density lipoprotein cholesterol and triglycerides (101). As lipid deposition progresses, localized oxidative stress and inflammation intensify, resulting in the depletion of nitric oxide and a consequent impairment of endothelium-dependent vasodilation (64, 100, 102, 103). This initial endothelial dysfunction serves as a pathogenic trigger, from which downstream disruptions across vascular, neural, endocrine, and immune systems subsequently ensue.

Unmitigated oxidative and inflammatory stress eventually forces structural remodeling of the corpus cavernosum. This environment promotes fibroblast activation and extracellular matrix deposition, culminating in progressive fibrosis (63, 104). Because these structural shifts reduce smooth muscle compliance, the veno-occlusive mechanism becomes mechanically compromised (105). Concurrent endocrine and neural dysregulation only compound this vascular decline. Therefore, the pathogenesis of dyslipidemia-associated ED involves numerous overlapping mechanisms.

## 9 Limitations

This review has some limitations. Most of the available evidence is indirect and based on systemic vascular, inflammatory, endocrine, and neural changes rather than studies directly performed in erectile ED. These mechanisms may help explain the association between dyslipidemia and ED, but they are not established causes. In humans, dyslipidemia frequently co-occurs with obesity, insulin resistance, hyperglycemia, and chronic inflammation, making it difficult to isolate its effects. Animal models also do not fully replicate human dyslipidemia, particularly regarding low HDL-C. Therefore, there is a clear need for ED studies that specifically address low HDL-C and distinguish the effects of lipids from other metabolic factors.

## 10 Conclusion and future perspectives

In summary, dyslipidemia may affect the male reproductive system through multiple interrelated mechanisms, including vascular injury, chronic inflammation, oxidative stress, neural dysregulation, and endocrine alterations, all of which can contribute to the development and progression of ED. Although the link between dyslipidemia and ED is increasingly recognized, understanding remains limited due to the complexity of its pathogenesis and the scarcity of direct, ED-specific evidence.

Future research should focus on several key directions. First, ED-specific preclinical and clinical studies are urgently needed to provide direct causal evidence and systematically elucidate the molecular mechanisms of dyslipidemia-induced ED. Particular emphasis should be placed on endothelial dysfunction-related signaling pathways, the impact of dyslipidemia on testosterone biosynthesis, regulation of cavernous nerve plasticity, and inflammation-driven fibrotic remodeling of the corpus cavernosum. These studies should also carefully distinguish the independent effects of dyslipidemia from confounding metabolic factors, including obesity, insulin resistance, hyperglycemia, and chronic low-grade inflammation, and clarify the specific role of low HDL-C in ED pathogenesis.

Second, mechanism-targeted therapeutic strategies should be systematically explored and validated using robust ED-specific experimental data. Additionally, integrating multimodal clinical data-such as lipid profiles, hormonal status, vascular imaging, and lifestyle factors-into machine learning-based risk prediction models may enable early identification of high-risk individuals and facilitate preemptive interventions. Such approaches hold substantial promise for improving quality of life and advancing lifelong health management.
